# Experimental evidence for glass polymorphism in vitrified water droplets

**DOI:** 10.1073/pnas.2108194118

**Published:** 2021-07-23

**Authors:** Johannes Bachler, Johannes Giebelmann, Thomas Loerting

**Affiliations:** ^a^Institute of Physical Chemistry, University of Innsbruck, Innrain 52c, A-6020 Innsbruck, Austria

**Keywords:** polyamorphism, glassy water, high-density amorphous ice, pressure-induced amorphization

## Abstract

The nature of amorphous ices has been debated for more than 35 years. In essence, the question is whether they are related to ice polymorphs or to liquids. The fact that amorphous ices are traditionally prepared from crystalline ice via pressure-induced amorphization has made a clear distinction tricky. In this work, we vitrify liquid droplets through cooling at ≥10^6^ K ⋅ s^−1^ and pressurize the glassy deposit. We observe a first order–like densification upon pressurization and recover a high-density glass. The two glasses resemble low- and high-density amorphous ice in terms of both structure and thermal properties. Vitrified water shows all features that have been reported for amorphous ices made from crystalline ice. The only difference is that the hyperquenched and pressurized deposit shows slightly different crystallization kinetics to ice I upon heating at ambient pressure. This implies a thermodynamically continuous connection of amorphous ices with liquids, not crystals.

## Significance

The question of whether a first-order liquid-to-liquid transition is at the origin of water’s anomalous properties has been controversial since the pioneering experiments by Mishima et al. in 1985 and molecular simulations by Poole et al. in 1992. Since then, experiments aimed at shedding light on this question have been performed using amorphous ices made from crystalline ice, fueling criticism about their crystal-like nature. In the present study, we avoid crystalline ice at any time of the experiment yet still observe a first-order glass-to-glass transition in vitrified liquid droplets. This makes the strong case for glass polymorphism and the direct thermodynamic connection to the liquid-to-liquid transition at higher temperatures, dismissing the criticism voiced for three decades.

## Two Liquids or a Mixture of Nanocrystals

**Fig. 1.**
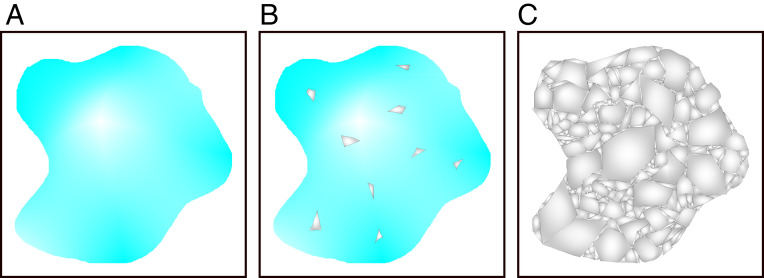
Three illustrations picturing HDA from different viewpoints, namely, (*A*) a pure glass, (*B*) a glassy matrix with nanocrystalline domains, or (*C*) a mixture of nanocrystallites. The figure is based on figure 2 in ref. 64.

Poole et al. (1) put forward the idea that the one-component system water could in fact be composed of two distinct liquids with differing densities (liquid polymorphism). Aimed at explaining the anomalous behavior of supercooled water, this model has remained in scientific debate for three decades (2–8). However, attempts to demonstrate the concept experimentally have not been conclusive because water readily crystallizes to ice in the temperature region where the two liquids could be separable. Hence, most experimentalists have resorted to study the amorphous forms, low-density amorphous ice (LDA) and high-density amorphous ice (HDA), which might represent glassy proxies of the two liquids (glass polymorphism or “poly-a-morphism”). The link between polyamorphism and liquid polymorphism hinges on the question of whether the amorphous ices are thermodynamically continuously connected with distinct liquids. This question has been controversial since the concept of glass polymorphism was coined by Mishima et al. (9). The key weakness in arguments favoring the two-liquid theory is that amorphous ices are commonly obtained starting from crystalline ices via pressure-induced amorphization (PIA) (9, 10). This has inspired the idea that amorphous ices are not related to liquids but represent distorted crystals (for a review on this topic, see ref. 11). The initial suggestion in favor of a two-liquid model by Mishima et al. (9) was that ice crystals experience thermodynamic ice melting followed by immediate transformation to the glassy state (vitrification). In this view, water ends up in HDA (see Fig. 1*A* for a simplified illustration). HDA and LDA can interconvert through an apparent first-order transition either by heating at low pressure or decompression (HDA → LDA) and compression at low temperatures (LDA → HDA) (12–14). Reversible and repeatable interconversion makes the case for glass polymorphism and is directly linked to liquid polymorphism in the view advocated by Mishima et al. This interpretation was challenged with the proposal that ice crystals experience mechanical melting upon compression (15). In this diametrical picture, HDA is viewed as a collection of collapsed, highly strained nanocrystals (see Fig. 1*C* for a simplified illustration) (16). These nanocrystallites are akin to high-pressure ice polymorphs, where both ice IV (17, 18) and ice VI (19) have been suggested as the polymorph to which HDA is similar. Consequently, the HDA↔LDA interconversion is interpreted as a polymorphic transition between distorted high-pressure crystals and distorted low-pressure ice I crystals in the diametrical view (15, 20–22). More recently, Seidl et al. attempted to harmonize the two-liquid model and the nanocrystal model by suggesting that HDA consists of a glassy matrix with embedded ice I nanocrystallites remaining after PIA (23) (see Fig. 1*B* for a simplified illustration). It was further suggested that the crystalline remnants disappear at sufficiently high pressure and temperature (24, 25). The procedure suggested by Seidl et al. is supposed to convert the amorphous ice from the raisin cake topology shown in Fig.1*B* to a homogeneous glass topology shown in Fig. 1*A*.

Considering the long-lasting debate, it is safe to say that additional experimental evidence is necessary to shed more light on the question. In this work, we study glass polymorphism but avoid starting from crystalline material. Our key question is whether a glass produced directly from the liquid features glass polymorphism or not. If this is the case, the two-liquid model is supported; if not, the view that the first-order transition in amorphous ices represents a transition between distorted ice polymorphs is fostered. To answer our key question, we start from stable liquid droplets and vitrify them using the hyperquenching technique invented in the 1980s by Brüggeller and Mayer (26). The glassy deposit, hyperquenched glassy water (HGW), is virtually identical to LDA in terms of structure and density (27, 28) yet offers the advantage that it is unambiguously connected to the liquid. The main feature distinguishing the present work from earlier work is recovery of the HGW deposit at 77 K from the vacuum chamber used for deposition and transfer to our high-pressure piston cylinder setup under liquid nitrogen. Compression experiments combined with X-ray and thermal data then allow us to answer our key question.

## HGW and its Behavior upon Compression

The current state of the art for producing HGW involves liquid micrometer-sized water droplets hitting at ultrasonic speed on a precooled copper plate (29). Resulting cooling rates ≥10^6^ K ⋅ s^−1^ lead to vitrified water of ≥95% purity. The remaining ≤5% may either be vitrified water or stacking-disordered ice I (ice I_sd_), where the error bar in the calorimetric method and the sensitivity of the diffraction measurements do not allow a distinction (30, 31). That is, HGW is possibly 100% glassy (as in Fig.1*A*) or possibly contains some small ice domains (as in Fig.1*B*). HGW experiences a subtle glass transition to a supercooled liquid of super-strong fragility (32) just before crystallization (31, 33, 34), which makes the case for a genuine glass (35). Calorimetric analysis based on the crystallization exotherm suggests that the purity of the HGW samples produced for this work is 96 ± 4%, thereby representing the state of the art (see *Thermal Behavior*).

**Fig. 2.**
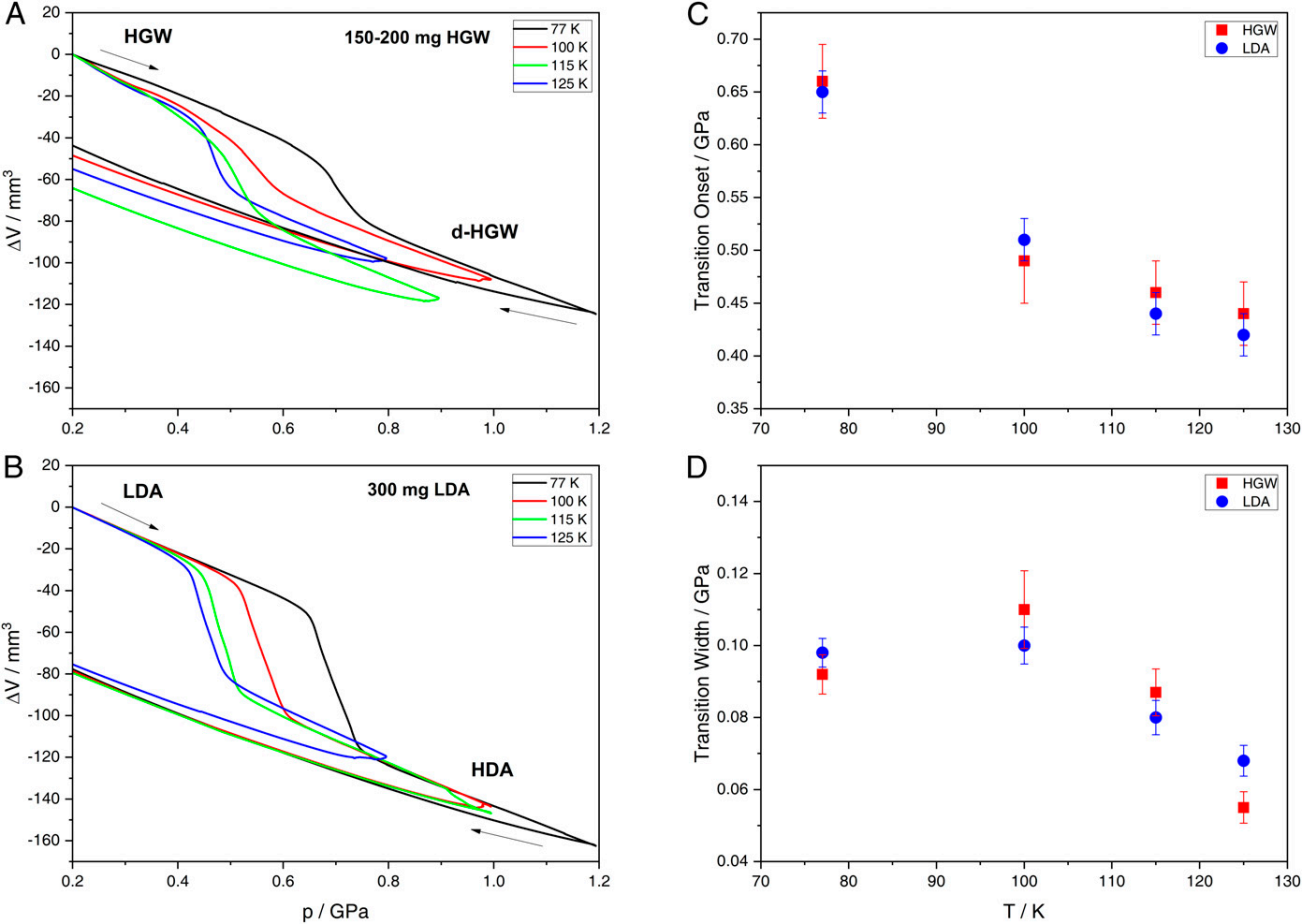
Volume change of (*A*) 150 to 200 mg HGW and (*B*) 300 mg LDA upon compression at 77 K (black), 100 K (red), 115 K (green), and 125 K (blue). (*C*) Onset pressure and (*D*) width of the transition in HGW (red) and LDA (blue) determined via the tangent method. Error bars represent ambiguities associated with tangent placement and determination of tangent intersections.

These glassy samples were then employed to search for glass-to-glass transformations under pressure. Fig. 2*A* shows the volume change of HGW upon compression at four different temperatures. At 77 K, HGW experiences an abrupt volume decrease (density increase) of 20 to 25% between 0.6 and 0.7 GPa. For comparison, amorphization of ice I occurs at much higher pressure, near 1.1 GPa (9, 10). That is, we here observe sudden densification of the glassy HGW matrix. At higher temperatures, this transformation gradually shifts to lower pressure, with an onset as low as 0.4 GPa at 125 K. For the purpose of a direct comparison, we have also prepared LDA samples starting from crystalline ice (13, 36). The compression curves of LDA are shown in Fig. 2*B*. The main difference is that for LDA samples, we are able to exactly define the weight of the sample in the bore (where 300 mg were used in this study), whereas it is not possible to weigh the HGW powder that was transferred under liquid nitrogen into the bore of the high-pressure cylinder. We estimate that we managed to transfer ∼150 to 200 mg of HGW into the cell. Nonetheless, the curves in Fig. 2*B* (LDA) are remarkably similar to the ones in Fig. 2*A* (HGW). On first look, it appears that the transition is sharper for LDA than it is for HGW. However, the evaluation of onset and offset pressure for the transformation using the tangent method disproves a smoother transition for HGW. Fig. 2*C* shows onset pressures and Fig. 2*D* the width of the transition (defined as offset pressure minus onset pressure). Both onset and width are identical for both samples within ±0.02 GPa. The volume change and the temperature dependence of onset pressure are fully consistent with the LDA→HDA transition reported previously (12, 37). That is, HGW experiences a sharp, possibly discontinuous, transformation to densified HGW (d-HGW), which might be the same as HDA. Another possibility would be that HGW rather crystallizes to a denser ice phase in a discontinuous way (e.g., to ice IX) (38). Such a behavior would be expected if the two-liquid model were incorrect and HGW represented the only genuine glassy form of water (20). These two scenarios are easily distinguished based on X-ray diffraction measurements as detailed in *Structural Information from X-Ray Diffraction*.

## Structural Information from X-Ray Diffraction

**Fig. 3.**
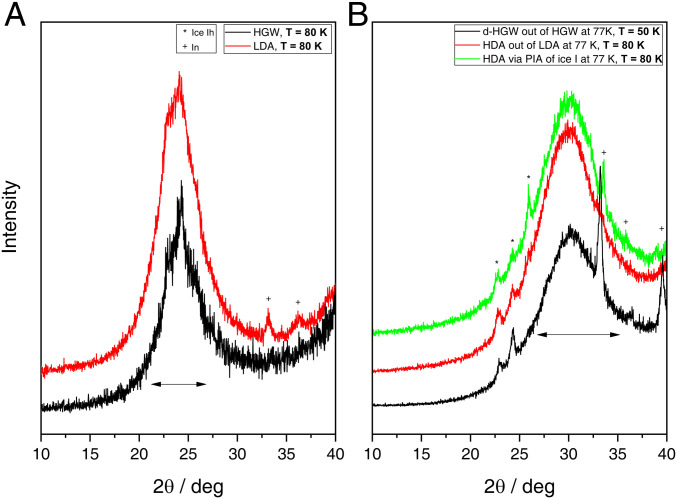
Powder X-ray diffractograms at 80 K. (*A*) HGW (black) and LDA (red). (*B*) d-HGW (densified at 77 K, black), HDA (made by PIA at 77 K, green), and HDA (made by compression of LDA at 77 K, red). Impurities of indium and ice I are marked by plusses and asterisks, respectively.

For the purpose of an ex situ X-ray analysis, the samples were cooled back to 77 K at high pressure to trap them in a kinetically arrested state and recovered by decompression to 1 bar (39). The smooth decompression curves in Fig. 2 *A* and *B* demonstrate that indeed no sharp transition occurs upon decompression, that is, the quench-recovered sample at 1 bar and 77 K represents the sample under high-pressure conditions. The X-ray patterns before and after compression are depicted in Fig. 3 *A* and *B*, respectively. LDA and HGW are known to be X-ray amorphous, both exhibiting an intense halo peak at the diffraction angle 2θ≈24° (31, 40), which is confirmed in Fig. 3*A* (2θ = 24.2 ± 0.2° for HGW and 2θ = 23.9 ± 0.2° for LDA). In addition, we find weak Bragg peaks pertaining to ice I in both amorphous solids. These mostly stem from condensation of water vapor onto the sample during the sample transfer process. After compression, the sample is still amorphous (black curve in Fig. 3*B*), again with traces of condensed humidity. However, the dominant halo peak is shifted from 2θ = 24.2 ± 0.2° (*d* = 0.368 ± 0.003 nm) to 2θ = 30.3 ± 0.2° (*d* = 0.295 ± 0.002 nm), indicating that HGW has transformed to a much denser amorphous state. That is, the sharp density jump in Fig. 2*A* originates from a glass-to-glass transition. This sharp glass-to-glass transition is the low-temperature counterpart to the first-order liquid-to-liquid transition. A very similar jump in halo peak position is also found after compression of LDA, yielding HDA, where the halo maximum is located at 2θ = 30.2 ± 0.2° (*d* = 0.296 ± 0.002 nm, red curve in Fig. 3*B*). Even HDA made by PIA of ice I (green curve in Fig. 3*B*) displays the halo peak maximum at this position, 2θ = 30.1 ± 0.2° (*d* = 0.297 ± 0.002 nm). That is, not only HGW and LDA but also d-HGW and HDA are indistinguishable based on X-ray diffraction. This is a very important result, especially considering that the same state is reached from very different starting points. This path independence also implies that the two glasses can be represented using a double well potential, where each glass corresponds to a minimum, which is metastable compared with a crystal. This is exactly the same energy landscape also found in simulations (42), most notably in the ST2 (Stillinger 2) water model (43). This suggests that also HDA prepared from crystalline material is glassy and can be understood in terms of Fig. 1 *A* or *B* but not in terms of Fig. 1*C*.

## Thermal Behavior

**Fig. 4.**
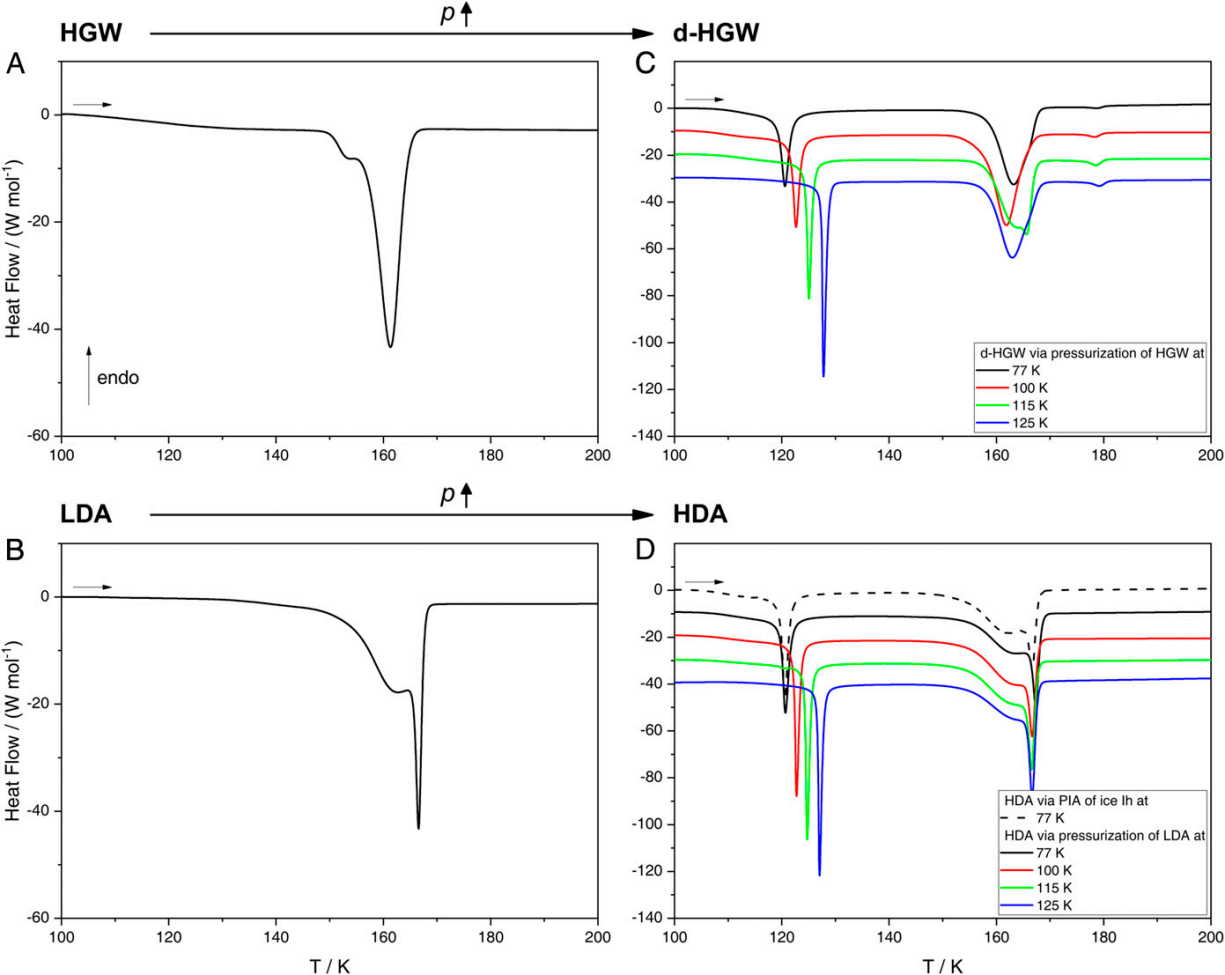
DSC warmup traces of (*A*) HGW, (*B*) LDA, (*C*) d-HGW, and (*D*) HDA recorded with a heating rate of 10 K/min. Scans in *C* and *D* are shifted vertically for clarity.

As a final step, we compare the thermal properties of these samples based on differential scanning calorimetry (DSC). The heating scans are shown in Fig. 4, and onset temperatures and transition enthalpies extracted from the scans are collected in Table 1. HGW (Fig. 4*A*) experiences a crystallization exotherm at 150 K with fronting, where a small shoulder precedes the strong exotherm. The exotherm releases a heat of −1.28 ± 0.04 kJ ⋅ mol^−1^, close to the literature value of −1.33 ± 0.02 kJ ⋅ mol^−1^ for 100% pure unannealed HGW (30). In other words, our HGW samples are in fact ∼96 ± 4% glassy. Also, the heating scan of LDA in Fig. 4*B* shows this fronting, where the shoulder is much more dominant, leading to a shift of the transition onset temperature to higher temperatures (cf. Table 1). We suggest that the asymmetry of the exotherm implies at least two types of crystallization kinetics, where the low-temperature kinetics is related to seeded growth of ice I and the high-temperature kinetics to nucleation and growth from the amorphous matrix. The more prominent shoulder in LDA then implies a greater ice I fraction in LDA that triggers earlier and faster crystallization of parts of the sample. The well-known but subtle glass transition of HGW and LDA at 136 K (33, 36) is masked by enthalpy relaxation in our scans.

**Table 1.** Onset temperatures (T onset) and transition enthalpies (ΔH**) **occurring in HGW/LDA and d-HGW/HDA, all prepared at 77 K, compared with literature (lit.) values (where available)

Transition	T onset, K	ΔH, kJ ⋅ mol^−1^	T onset (lit.), K	ΔH (lit.), kJ ⋅ mol^−1^	Rate, K ⋅ min^−1^	Ref.
HGW → ice I_sd_	156.3 ± 0.5	−1.28 ± 0.04	158.5 ± 0.2	−1.33 ± 0.02	10	30
LDA → ice I_sd_	165.5 ± 0.8	−1.30 ± 0.02	143.1 ± 0.1	−1.36 ± 0.01	0.17	45
			∼156	−1.37 ± 0.06	30	36
			∼165	−1.27 ± 0.06	10	44
d-HGW (77 K) → HGW’	118.7 ± 0.6	−0.50 ± 0.02	—	—	—	—
HDA (77 K) → LDA	119.7 ± 0.4	−0.53 ± 0.06	113.4 ± 0.1	−0.515 ± 0.005	0.17	45

Errors are given as SDs, where 5/5/6/5 scans were collected for HGW/LDA/d-HGW/HDA. The onset temperatures of ref. 36 and 45 are estimated from the graphs presented therein. Please note that the complete set of calorimetric data obtained for this work is available in the *SI Appendix*.

After densification, the thermal behavior of HGW changes substantially. d-HGW prepared at 77 K shows a pronounced exotherm at 119 K that is not present in unpressurized samples (black line in Fig. 4*C*). The same peak is found for HDA made by PIA (dashed line in Fig. 4*D*) as well as for HDA made by compression of LDA at 77 K (black line in Fig. 4*D*). In the latter two cases, this exotherm is known to indicate the polyamorphic transition from HDA to LDA (13), from one minimum to the other in the metastable double-well potential. That is, this exotherm signifies the sharp transition from one glassy state to another. There is no reason to interpret the exotherm at 119 K appearing in d-HGW samples in any other way. d-HGW is thermally the same as HDA. This becomes even more evident when inspecting the impact of preparation temperature: An increase of temperature from 77 to 125 K for the compression process (Fig. 2 *A* and *B*) shifts the polyamorphic transition observed at ambient pressure from 119 to 127 K in both cases (Fig. 4 *C* and *D*). This can be rationalized based on relaxation effects, where a more relaxed, deeper HDA state (closer to the minimum in the well) is attained under pressure at higher temperatures (24). The deeper HDA then requires more thermal energy (i.e., higher temperatures) in the calorimetry experiment to cross the activation barrier and to reach the second glassy state, LDA (43). The observation of the HDA → LDA exotherm makes the unique case for “glass polymorphism.” Exotherms are typical of first-order transitions and represent the heat released in the transition. The observation of such exotherms for transitions between two glasses is spectacular because glassy water is intrinsically out of equilibrium in nature yet shows heat release typical of a discontinuous phase transition governed by equilibrium thermodynamics. In other words, the transition between HDA and LDA is an activated transition in a double-well potential that can be accessed in experiments as if the more stable crystalline phase were nonexistent. This distinguishes the example of glassy water from most other oxidic glasses featuring “simple” relaxation. The congruence of the thermal signatures for HDA and d-HGW advocates the view that HDA is just as glassy as d-HGW, but is not a mixture of collapsed ice crystals.

Interestingly, the shape of the second exotherm—the crystallization exotherm near 160 K—differs considerably between samples that were hyperquenched and densified (Fig. 4*C*) and ones that were pressure-amorphized and densified (Fig. 4*D*). While HDA samples often show a pronounced shoulder at the peak front at 145 K (Fig. 4*D*) (44), d-HGW samples do not (Fig. 4*C*). Furthermore, d-HGW samples exhibit a small exotherm corresponding to the transformation from stacking-disordered ice (ice I_sd_) to hexagonal ice (ice I_h_) at 180 K, contrasting the case of HDA where it is reported at 225 K (45). We interpret this in the sense that seeding with ice I (see Fig. 1*B*) plays a key role (46), where the seeds present in the glassy matrix differ in these two types of preparation routes. Some ice I might be present after hyperquenching because, locally, not all the heat is carried away. These ice I_sd_ seeds are retained upon compression to d-HGW. By contrast, HDA samples prepared through PIA contain distorted, nanocrystalline ice I remnants (different from ice I_sd_) because of incomplete amorphization. The seeds in HGW are more efficient to grow ice I_sd_—giving rise to an earlier onset and different shape of the crystallization exotherm. Furthermore, this also produces ice I that differs in terms of “cubicity” (i.e., the fraction of cubic stacking faults in the hexagonal stacking sequence) (47, 48). Cubicity seems to be quite low for ice I from d-HGW (see *SI Appendix*, Figs. S1 and S2 for X-ray diffraction data consistent with this idea). Consequently, also the weak exotherm indicating transformation from ice I (containing cubic stacking faults) to ice I_h_ (containing hexagonal stacks only) is shifted in the calorimetry scans. While the crystallization exotherm and the polytypic conversion to hexagonal ice are strongly affected through the different ice I impurities in the amorphous matrix, the glass-to-glass exotherm is not at all affected by the presence of these impurities. This makes sense because crystalline impurities do not impede the growth of a glass of low density inside a glassy matrix of high density. In fact, Tonauer et al. have shown that this process involves nucleation and growth of LDA domains within HDA as well as interfaces between LDA and HDA (49). Also, these observations are typical of truly discontinuous transitions and show that “glass polymorphism” is governed by thermodynamics and not by the kinetics of relaxation.

## Compatibility with Water Models

**Fig. 5.**
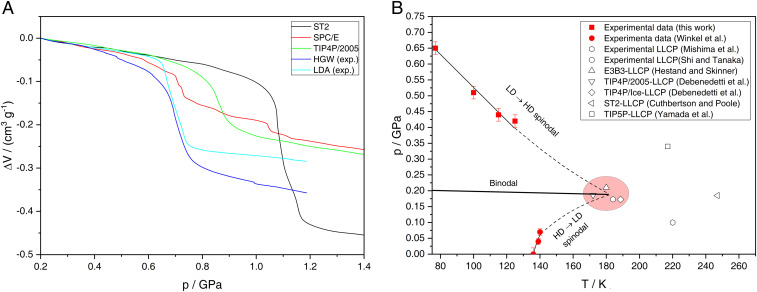
Comparison of our results with data from literature. (*A*) Volume change of glassy ST2 from ref. 51, SPC/E from ref. 53, TIP4P/2005 water from ref. 52, and HGW/LDA from our experiments. All curves are aligned at 0.2 GPa for better comparison. A blind experiment was subtracted from the LDA and HGW curves. (*B*) Experimental estimates of the low-to-high and high-to-low–density spinodals based on onset pressures from Fig. 2 (LD → HD; red squares) and from ref. 40 (HD→LD; red filled circles). Estimates for liquid-to-liquid critical points are shown as open symbols; for “real” water from ref. 65 (hexagon) and ref. 57 (circle); and for water models from ref. 66 for E3B3 (upward triangle), ref. 56 for TIP4P/2005 (downward triangle) and TIP4P/Ice (diamond), ref. 67 for ST2 (left triangle), and ref. 68 for TIP5P (square). The dashed lines are extrapolations of our measured spinodals to the critical point (light red ellipse).

Finally, our results allow for direct comparison with molecular dynamic simulations employing full-atomistic water models. This was not possible so far because LDA in experiments is usually prepared starting from ice I_h_, while in simulations, “LDA” (in fact, HGW) is prepared through ultrarapid cooling of liquid water (2). By contrast to experiments, vitrification is realized easily in simulations because it is rare to observe unseeded crystallization of water. Consequently, experimentalists often have to choose PIA of crystalline ice as the path to amorphous ices. Our study benefits from a powerful hyperquenching setup that has been invented in Innsbruck (29) and improved over 30 y to reach cooling rates as high as 10^7^ K ⋅ s^−1^ (31), very close to the lowest cooling rates employed in simulations (50). This vitrification setup, combined with the procedure of transferring hyperquenched deposits to our high-pressure cell provides the opportunity to follow the exact same thermodynamic path in experiments that was also followed in many simulations in the past (50–55). In Fig. 5*A*, we compare compression behavior of HGW and LDA in experiment with several water models in simulation (ST2, SPC/E, and TIP4P/2005) at 77 to 80 K. Notably, there are pronounced differences between the different water models: ST2 (black) exhibits an abrupt transition from low- to high-density glass at about 1.0 GPa, involving an exaggerated densification of ∼75% (51). There is no sharp transition in SPC/E (red) but rather continuous densification starting at ∼0.6 GPa (53). These discrepancies are due to overemphasizing the tetrahedral bonding motif of H_2_O molecules in ST2 and underrepresenting it in SPC/E. For TIP4P/2005 water, the most realistic water model of the three, a clear glass-to-glass transition is observed at 0.80 GPa and 80 K (green) (52, 54). The recovered high-density glass exhibits a densification of 25% consistent with our experiments (light blue and blue). However, the transition pressure deviates from the experimental value of ∼0.6 GPa for HGW/LDA. This is most likely due to the fact that the onset pressure is quite sensitive to the compression rate, occurring at 0.80 GPa for 10^10^ MPa ⋅ s^−1^ (50, 52, 54) and at 0.75 GPa for 10^8^ MPa ⋅ s^−1^ (50) in simulations. Extrapolating the onset pressure in simulations to experimental compression rates of ∼3 MPa ⋅ s^−1^ yields ∼0.6 GPa, very close to what we observe in experiments (50). That is, out of the three models, TIP4P/2005 water reproduces the glass polymorphism behavior of real hyperquenched water best. Notably, it was recently demonstrated that this model shows a clear liquid-to-liquid critical point at 172 K and 0.186 GPa (56). Our experiments do not probe for the existence of the liquid-to-liquid critical point directly. Yet, the temperature dependence of the low- to high-density transitions observed in Fig. 2 allows us to define part of the spinodal line emerging from the critical point. In Fig. 5*B*, the onset pressures extracted from Fig. 2 are depicted as red squares, where the experimental spinodal determined from these points is drawn as a solid black line. The red circles in Fig. 5*B* are data points from our earlier work (40) that allow us to define a small portion of the high- to low-density spinodal. The dashed black lines in Fig. 5*B* are extrapolations to ∼180 K and ∼0.2 GPa, where the critical point is located for several models (see open symbols in Fig. 5*B*), including TIP4P/2005, TIP4P/Ice, E3B3, and the two-state model designed by Shi and Tanaka (57). We do not claim existence of the second critical point based on our experiments. Yet, if it exists, our spinodals favor a low-lying critical point near 180 K (marked by red ellipses in Fig. 5*B*) over critical points at higher temperatures.

## Summary and Conclusion

The key question in this work is whether a glass produced directly from the liquid features glass polymorphism or not. Our results provide a clear affirmative answer, demonstrating that vitrified water droplets undergo a first order–like transition upon compression. d-HGW is just like HDA in terms of X-ray diffraction and thermal properties. That is, it also shows a diffuse X-ray pattern with the halo peak maximum at the diffraction angle of 2θ ≈ 30° at 80 K and an apparent-first order transition to a low-density state where the onset is highly sensitive to compression temperature. The calorimetric observation of a sharp exotherm is a hallmark for an apparent first-order transition from one glass to a second, distinct glass despite glasses being in nonequilibrium. This glass-to-glass transition makes the case for glass polymorphism, which is a fundamental prerequisite for the two-liquid model of water. The equivalence of HDA to d-HGW and LDA to HGW is incompatible with the idea of HDA and LDA being distorted crystals in nature. Consequently, we claim that PIA at 77 K (or higher) can be explained as a thermodynamic melting event but not as a mechanical collapse of ice I. This is in agreement with the conclusions by Strässle et al., who show that ice I_h_ experiences mechanical collapse only for lower temperatures (58). Furthermore, our work provides the thermodynamic link for the observations by Mishima and Suzuki, who quenched pressurized and emulsified water (59). The quite blurry X-diffractograms interpreted as “HDA” by them are indeed equivalent to a densified glass (i.e., d-HGW introduced here). That is, d-HGW (or HDA) can both be reached by compression of HGW (or LDA) or through quenching of the pressurized liquid. In other words, all thermodynamic links between liquid water and amorphous ices, including the glass polymorphism link, have now been demonstrated experimentally. Even the last remaining link, the direct observation of the liquid-to-liquid transition above the glass transition temperature, seems to have been found: Winkel et al. suggested that they observed a first-order transition between two ultraviscous liquids slightly above the two glass transition temperatures of LDA and HDA through decompression at 140 K (14). Very recently, Kim et al. found evidence for a liquid-to-liquid transition by ultrafast heating of HDA to about 205 K (i.e., far above the glass transition), where bulk water rapidly crystallizes (60). At about the same time, Kringle et al. locate the transition from high- to low-density states in the range from 245 to 190 K, supporting the idea of a two-state model based on vacuum experiments (61). While strongly supporting the two-liquid model of water, our results do not imply whether two-liquid coexistence terminates in a liquid-to-liquid critical point (1, 41, 56) or not (62). From compression behavior alone, we find that the best fit to experimental data is provided by the TIP4P/2005 model, which exhibits a liquid-to-liquid critical point (56). The location of this critical point is compatible with our rough estimate based on extrapolation of experimentally determined spinodals to higher temperature.

## Materials and Methods

For hyperquenching, we employed the setup of Kohl et al. (31). In brief, droplets ∼3 µm in diameter made from Milli-Q water were produced using an ultrasonic nebulizer and conveyed into a high-vacuum system through a 300-µm aperture with nitrogen as the carrier gas. The droplets were then deposited on an oxygen-free highly conductive copper substrate precooled to ≤80 K. After 30 min of deposition, an HGW layer ∼2- mm thick was obtained. The vacuum was broken with dry nitrogen gas, and the substrate was quickly plunged into liquid nitrogen. While remaining in liquid nitrogen, HGW was scratched off the copper substrate mechanically, encapsulated in a cylindrical indium container, and transferred into the bore of a steel piston cylinder setup. Indium is necessary to avoid shockwave heating due to friction upon compression (9). We used a material testing machine by Zwick Roell (model BZ100/TL3S), which applies force vertically. In addition, the machine records the piston position with spatial resolution of 0.01 µm. Samples were precompressed slowly to 0.2 GPa at 77 K to squeeze air out of the container and brought back to ambient pressure. This leads to a change in slope at 0.2 GPa during subsequent recompression. Therefore, the curves are shown only at pressures above 0.2 GPa in Fig. 1, while the complete cycles are found in *SI Appendix*, Figs. S3 and S4. After the compression cycles at various temperatures with compression rates of 200 MPa/min (shown in Fig. 1), samples were quenched, brought to ambient pressure, and recovered. Weighing the sample to deduce the mass of employed HGW is not possible because of rapidly evaporating liquid nitrogen. As a remedy, we have used the height of the recovered cylinder (7-mm diameter) for a rough estimate. This way, we determined the amount of HGW in each compression cycle to be around 150 to 200 mg.

Reference LDA and HDA samples were prepared entirely in the piston cylinder setup. First, 300-µL water at room temperature was pipetted into an indium cylinder precooled to 77 K. This yields ice I_h_, which is compressed to 1.6 GPa at 77 K, causing transformation to HDA. LDA, or more specifically LDA-I (28), was then obtained by isobaric heating of HDA to 140 K at 0.01 GPa (63). The ensuing compression procedure (including precompression) is identical to the one described above for HGW samples.

Ex situ X-ray diffraction is performed on a D8 Bruker Advance X-ray diffractometer with incident wavelength λ = 0.154178 nm (CuK-α). The instrument is equipped with a Goebel mirror, *LynxEye XE-T* array detector, and a low-temperature chamber by FMB Oxford Ltd. Accurate temperature control between 20 and 300 K is possible by combining a Si-diode with a two-stage helium cryostat and resistive heating elements. Recovered HGW/d-HGW samples are rather sticky and difficult to transfer into the instrument without significant amounts of contamination. Therefore, we deposited HGW on a self-designed copper substrate that functions as a sample holder and is easily transferrable to our instrument precooled to ∼70 K. On the other hand, for d-HGW, the copper sample holder was mounted in the instrument and again precooled to ∼70 K before the whole indium container was inserted into the bore of the sample holder. Other samples are finely powdered under liquid nitrogen and transferred onto another sample holder designed for powders specifically.

Ex situ DSC was carried out on a DSC 8000 by PerkinElmer. Temperature calibration was done using cyclopentane, adamantane, and indium. About 15 mg of sample was transferred into aluminum crucibles and cold-loaded into the instrument. The samples were warmed from 93 to 298 K, cooled, and again reheated, all with rates of 10 K ⋅ min^−1^. The second heating trace serves as a baseline. The scans were normalized using the melting peak where the value of 6.008 kJ ⋅ mol^−1^ was used as melting enthalpy of ice.

## Supplementary Material

Supplementary File

## Data Availability

All study data are included in the article and/or *SI Appendix*.
