# Supplementary material for: Experimental evidence for glass polymorphism in vitrified water droplets
Source: Proc Natl Acad Sci U S A. 2021 Jul 23;118(30):e2108194118. doi: 10.1073/pnas.2108194118 (PMC8325285; doi:10.1073/pnas.2108194118)
Supplement: Supplementary File [file pnas.2108194118.sapp.pdf]

Supporting Information for:

"Experimental evidence for glass polymorphism in vitrified water droplets"

Johannes Bachler, Johannes Giebelmann, Thomas Loerting*

Institute of Physical Chemistry, University of Innsbruck, Innrain 52c, A-6020 Innsbruck, Austria

This file contains temperature-dependent powder X-ray diffraction (XRD) scans of d-HGW and HDA, tabulated calorimetric data of all scans shown in Fig. 4 and the complete volume curves of Fig. 2.

Temperature-dependent X-ray diffraction experiments

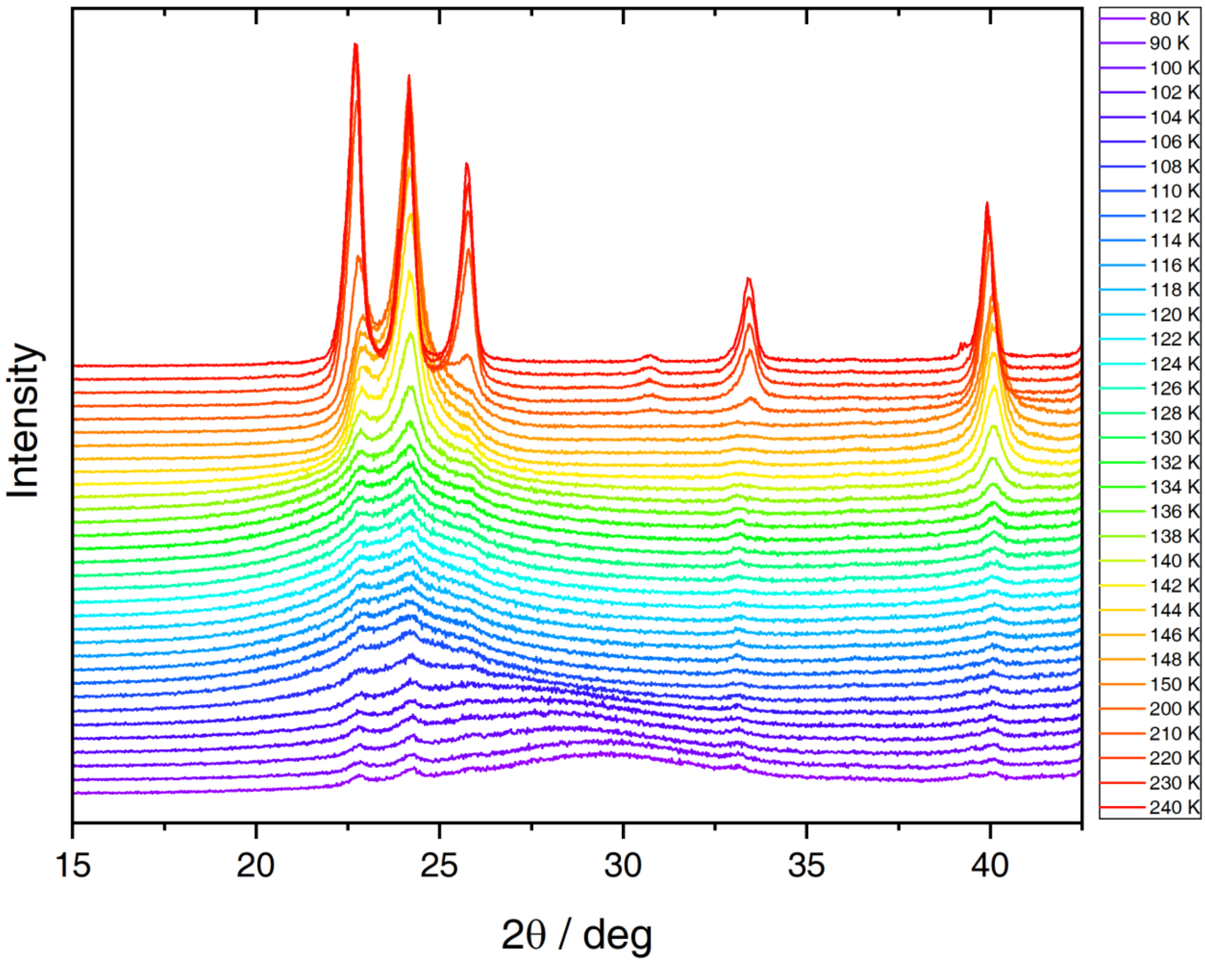

Fig. S1. X-ray diffractograms of d-HGW made by compression of HGW at 77 K recorded between 80 and 240 K. These scans show a greater ice I contamination than the scan presented in Fig. 3b of the main manuscript because they were carried out on a finely powdered d-HGW sample to ensure good thermal contact with the sample holder.

Fig. S1 shows temperature dependent XRD scans of d-HGW made by compression of HGW at 77 K and Fig. S2 shows temperature-dependent XRD scans of HDA made by compression of LDA at 77 K. The curves at 80 K show the pronounced diffuse halo peak pattern of the high-density polyamorph at $2\theta \approx 30°$ as well as slight contaminations of ice I_h due to the sample transfer procedure. Most notably, at ≈ 110 K the position of the halo peak suddenly shifts from $2\theta \approx 30°$ to $2\theta \approx 24°$, which is in remarkable agreement with the polyamorphic transition inferred *via* DSC. That is, also the structural behavior of d-

HGW and HDA with temperature is highly similar. Cold-crystallization to ice I_{sd} sets in at ≈140 K. Here the intensity of the peak at 2θ≈26° relative to the ones at 2θ≈22 and 24° is somewhat lower in d-HGW samples than in HDA samples, hinting that d-HGW crystallizes to a more stacking-disordered form of ice I than HDA. The transition to stable ice I_h occurs at ≈200 K.
We note that the lower transition temperatures in the XRD instrument compared to the DSC measurements of Figure 3 are due to significant differences in heating rates: In our XRD chamber, each temperature is approached with ≤6 K/min where each individual scan consumes additional 30 min. This results in an effective heating rate of ≤0.14 K/min, substantially lower than the 10 K/min employed in DSC.

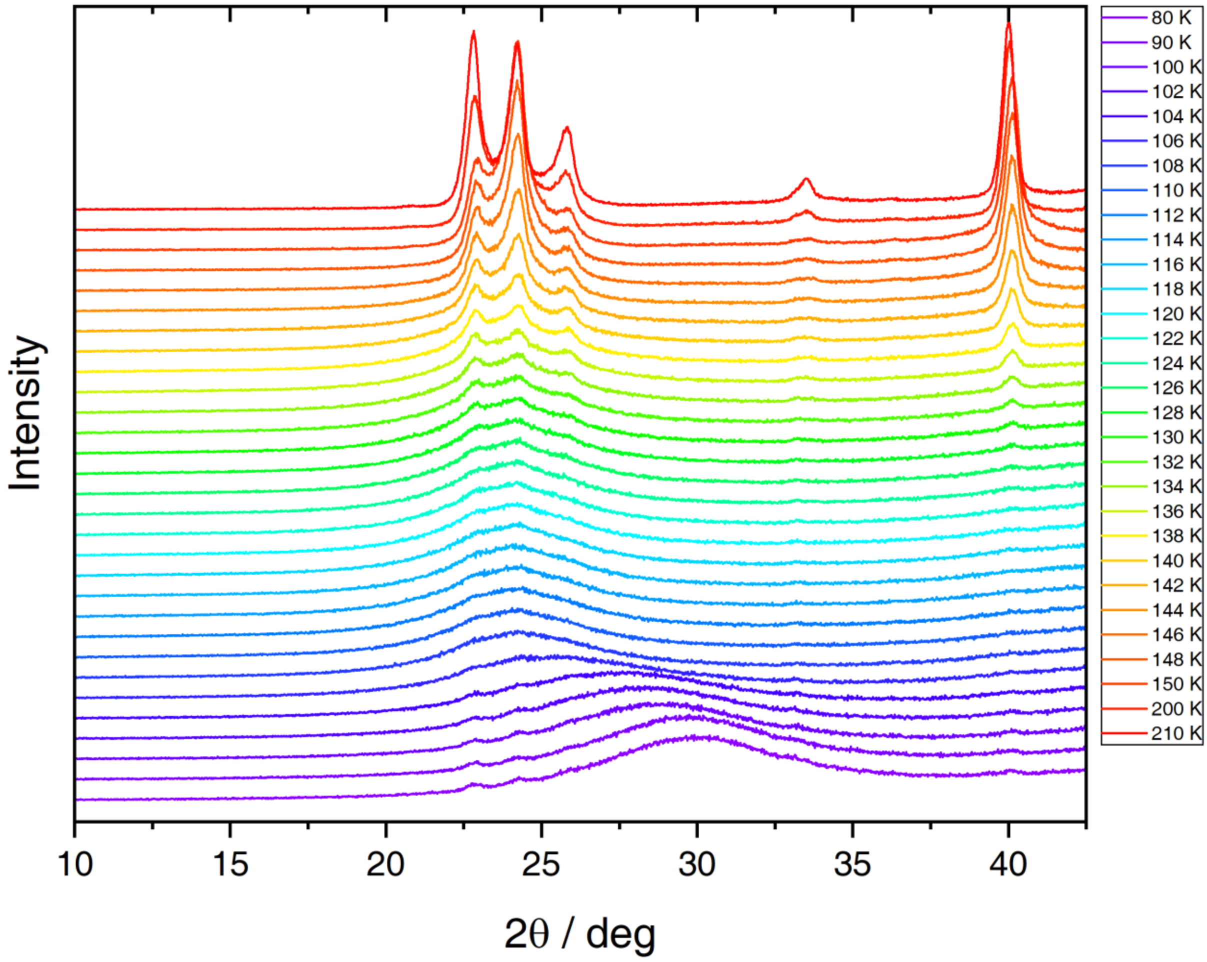

Fig. S2. X-ray diffractograms of HDA made by compression of LDA at 77 K recorded between 80 and 210 K.

Calorimetric data

Tab. S1-S4 collect calorimetric data, i.e., onset temperatures, minimum temperatures (positions of the exothermic peaks) and transition enthalpies with errors (given as standard deviation) for all scans displayed in Fig. 4 of the main manuscript. d-HGW samples obtained after compression at 115-125 K differ from samples compressed at 77-100 K. The latent heat evolved for the polyamorphic transition and the cold-crystallization to ice I_{sd} are both smaller for the former than for the latter. This is due to partial crystallization of the sample and frictional heating during compression.

Tab. S1. Thermal data for HGW.

Deposition T / K	Run No.	Transition	Onset T / K	Minimum T / K	ΔH / kJ mol^{-1}
77	1	HGW → ice I_{sd}	156.4	160.8	-1.30
77	2	HGW → ice I_{sd}	155.4	160.4	-1.28
77	3	HGW → ice I_{sd}	156.6	160.8	-1.28
77	4	HGW → ice I_{sd}	156.8	161.3	-1.33
77	5	HGW → ice I_{sd}	156.3	160.9	-1.22
**77**	**Mean**	**HGW → ice I_{sd}**	**156.3±0.5**	**160.8±0.3**	**1.28±0.04**

Tab. S2. Thermal data for HDA prepared by compression of ice I (PIA).

Compression T / K	Run No.	Transition	Onset T / K	Minimum T / K	ΔH / kJ mol^{-1}
77	1	HDA → LDA	119.7	120.6	-0.544
77	2	HDA → LDA	119.2	120.3	-0.592
77	3	HDA → LDA	120.2	121.4	-0.663
77	4	HDA → LDA	119.8	120.5	-0.590
77	5	HDA → LDA	119.5	120.5	-0.622
**77**	**Mean**	**HDA → LDA**	**119.7±0.4**	**120.7±0.4**	**0.60±0.04**
77	1	LDA → ice I_{sd}	164.9	166.6	-1.28
77	2	LDA → ice I_{sd}	164.7	166.5	-1.30
77	3	LDA → ice I_{sd}	166.7	168.5	-1.31
77	4	LDA → ice I_{sd}	166	167.2	-1.27
77	5	LDA → ice I_{sd}	165.3	166.6	-1.32
**77**	**Mean**	**LDA → ice I_{sd}**	**165.5±0.8**	**167.1±0.8**	**1.30±0.02**

Tab. S3. Thermal data for d-HGW prepared by compression of HGW.

Compression T / K	Run No.	Onset T / K	Minimum T / K	ΔH / kJ mol^{-1}
**Glass–glass transition**				
77	1	119.5	120.6	-0.488
77	2	119.1	120.5	-0.536
77	3	118.1	120.1	-0.476
77	4	118.5	120.4	-0.493
77	5	119.0	120.4	-0.488
77	6	118.1	120.2	-0.492
**77**	**Mean**	**118.7±0.6**	**120.4±0.2**	**-0.50±0.02**
100	1	121.6	122.7	-0.561
100	2	121.6	122.6	-0.524
**100**	**Mean**	**121.6**	**122.7±0.1**	**-0.54±0.03**
115	1	124.4	125.1	-0.444
115	2	125.1	125.8	-0.526
115	3	125.7	126.5	-0.538
115	4	124.7	125.4	-0.503
**115**	**Mean**	**125.0±0.6**	**125.7±0.6**	**-0.50±0.04**
125	1	127.3	128.1	-0.441
125	2	127.2	127.8	-0.425
125	3	127.9	128.5	-0.404
125	4	127.3	127.7	-0.543
125	5	127.2	127.9	-0.490
**125**	**Mean**	**127.4±0.3**	**128.0±0.3**	**-0.46±0.06**
**Crystallization to ice I_{sd}**				
77	1	161.5	164.4	-1.17
77	2	158.5	163.2	-1.17
77	3	156.4	162.2	-1.26
77	4	157.7	162.8	-1.23
77	5	159.7	163.1	-1.23

77	6	156.9	162.4	-1.25
**77**	**Mean**	**158±2**	**163.0±0.8**	**-1.22±0.04**
100	1	157.7	161.9	-1.30
100	2	157.5	162.1	-1.31
**100**	**Mean**	**157.6±0.1**	**162.0±0.1**	**-1.31±0.01**
115	1	158.2	165.7	-1.13
115	2	159.4	164.6	-1.12
115	3	160.0	164.2	-1.25
115	4	158.1	162.6	-1.22
**115**	**Mean**	**158.9±0.9**	**164.3±1.3**	**-1.18±0.07**
125	1	157.2	162.1	-0.983
125	2	156.8	161.8	-0.978
125	3	158.1	162.9	-0.926
125	4	158.4	162.9	-1.15
125	5	157.8	163.0	-1.14
**125**	**Mean**	**157.7±0.7**	**162.5±0.6**	**-1.03±0.10**

**Polytypic ice $I_{sd} \rightarrow I_h$ transition**				
77	1	177.2	179.4	-0.010
77	2	176.8	178.7	-0.008
77	3	176.2	178.0	-0.018
77	4	177.3	179.1	-0.015
77	5	177.8	180.0	-0.019
77	6	176.7	178.3	-0.018
**77**	**Mean**	**177.0±0.6**	**178.9±0.7**	**-0.015±0.005**
100	1	176.3	178.4	-0.020
100	2	178.2	178.2	-0.017
**100**	**Mean**	**177±1**	**178.3±0.1**	**-0.019±0.002**
115	1	176.5	178.6	-0.036
115	2	178.0	180.1	-0.021
115	3	178.8	181.1	-0.026
115	4	176.5	178.9	-0.034
**115**	**Mean**	**177.5±1.1**	**179.7±1.2**	**-0.029±0.007**
125	1	177.2	178.9	-0.041
125	2	176.2	178.3	-0.051
125	3	178.1	179.8	-0.029
125	4	177.4	179.2	-0.022
125	5	177.0	179	-0.027
**125**	**Mean**	**177.2±0.7**	**179.0±0.5**	**-0.034±0.012**

Tab. S4. Thermal data for HDA prepared by compression of LDA.

Compression T / K	Run No.	Onset T / K	Minimum T / K	ΔH / kJ mol^{-1}
**Polyamorphic HDA → LDA transition**				
77	1	119.7	120.7	-0.56
100	1	122.2	122.8	-0.552
100	2	122.4	123.0	-0.647
100	3	122.0	122.7	-0.577
**100**	**Mean**	**122.2±0.2**	**122.8±0.2**	**-0.59±0.05**
115	1	124.2	124.8	-0.565
115	2	124.6	125.1	-0.529
**115**	**Mean**	**124.4±0.3**	**125.0±0.2**	**-0.55±0.03**
125	1	127.0	127.5	-0.496
125	2	126.5	127.0	-0.503
125	3	126.9	127.6	-0.446
**125**	**Mean**	**126.8±0.3**	**127.4±0.3**	**-0.48±0.03**
**Crystallization to ice I_{sd}**				
77	1	165.8	167.3	-1.16
100	1	165.1	166.7	-1.29
100	2	165.9	167.4	-1.39
100	3	165.6	167.0	-1.29
**100**	**Mean**	**165.5±0.4**	**167.0±0.4**	**-1.32±0.06**
115	1	165.1	166.6	-1.26
115	2	166.0	167.4	-1.22
**115**	**Mean**	**165.6±0.6**	**167.0±0.6**	**-1.24±0.03**
125	1	165.3	166.9	-1.07
125	2	165.3	166.6	-1.15
125	3	165.2	167.3	-0.976
**125**	**Mean**	**165.3±0.1**	**166.9±0.4**	**-1.07±0.09**

Raw volume curves

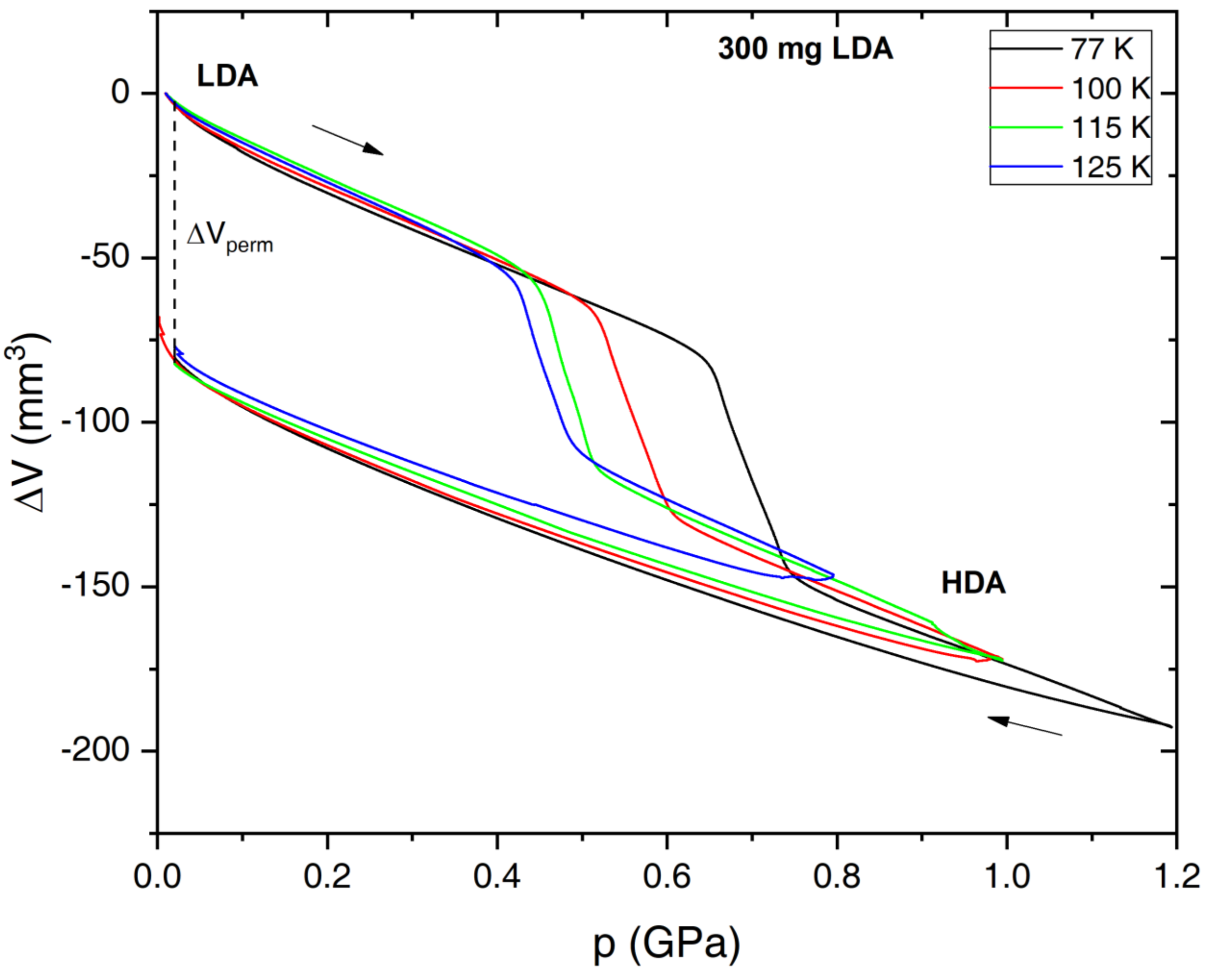

Fig. S3. Volume change behavior of 300 mg LDA samples upon compression at 77 (black), 100 (red), 115 (green) and 125 K (blue). The permanent volume change ΔV_{perm} indicated by the dashed line at 0.02 GPa comprises densification of the sample and densification of the machine itself, e.g., steel pistons. The blind experiment measuring the machine contribution has been subtracted in Fig. 5 of the main manuscript but not in Fig. 2.

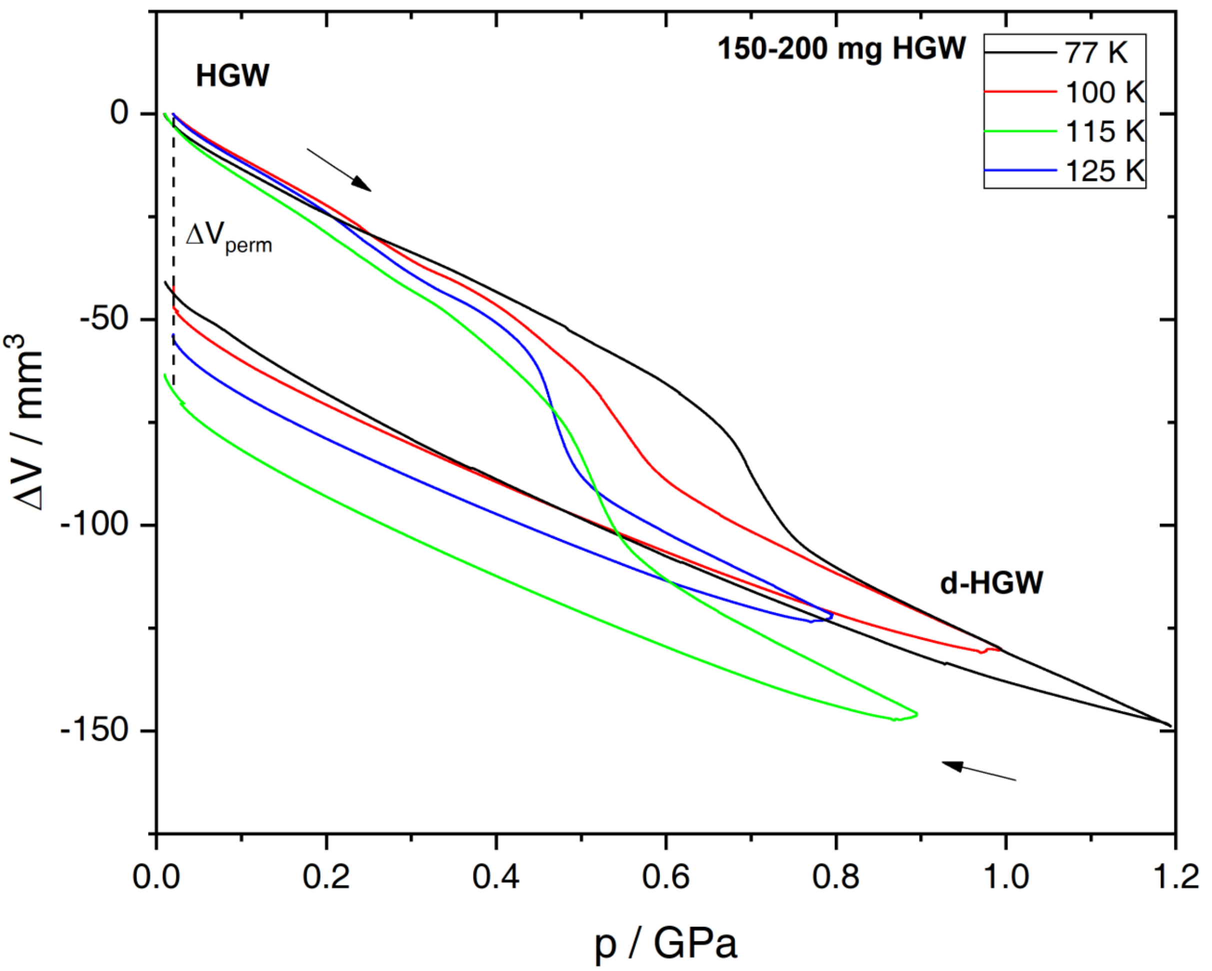

Figure S4. Volume change behavior of 150-200 mg HGW samples upon compression at 77 (black), 100 (red), 115 (green) and 125 K (blue). Again, the blind experiment measuring the machine contribution has been subtracted in Fig. 5 of the main manuscript but not in Fig. 2. ΔV_{perm} is differing in all three curves due to different amounts of sample employed in each experiment.
